# Genetic Relationships, Carbendazim Sensitivity and Mycotoxin Production of the *Fusarium Graminearum* Populations from Maize, Wheat and Rice in Eastern China

**DOI:** 10.3390/toxins6082291

**Published:** 2014-08-04

**Authors:** Jianbo Qiu, Jianrong Shi

**Affiliations:** Key Laboratory of Food Quality and Safety of Jiangsu Province—State Key Laboratory Breeding Base; Key Laboratory of Control Technology and Standard for Agro-product Safety and Quality (Nanjing), Ministry of Agriculture; Key Lab of Agro-product Safety Risk Evaluation (Nanjing), Ministry of Agriculture; Institute of Food Quality and Safety, Jiangsu Academy of Agricultural Sciences, Nanjing 210014, Jiangsu, China; E-Mail: qiujianbo19850901@126.com

**Keywords:** *Fusarium graminearum*, trichothecene chemotype, genetics relationships, mycotoxin production, carbendazim resistance

## Abstract

Members of the *Fusarium graminearum* species complex (FGSC) are important pathogens on wheat, maize, barley, and rice in China. Harvested grains are often contaminated by mycotoxins, such as the trichothecene nivalenol (NIV) and deoxynivalenol (DON) and the estrogenic mycotoxin zearalenone (ZEN), which is a big threat to humans and animals. In this study, 97 isolates were collected from maize, wheat, and rice in Jiangsu and Anhui provinces in 2013 and characterized by species- and chemotype-specific PCR. *F. graminearum sensu stricto* (s. str.) was predominant on maize, while most of the isolates collected from rice and wheat were identified as *F. asiaticum. Fusarium* isolates from three hosts varied in trichothecene chemotypes. The 3-acetyldeoxynivalenol (3ADON) chemotype predominated on wheat and rice population, while 15ADON was prevailing in the remaining isolates. Sequence analysis of the translation elongation factor 1α and trichodiene synthase indicated the accuracy of the above conclusion. Additionally, phylogenetic analysis suggested four groups with strong correlation with species, chemotype, and host. These isolates were also evaluated for their sensitivity to carbendazim and mycotoxins production. The maize population was less sensitive than the other two. The DON levels were similar in three populations, while those isolates on maize produced more ZEN. More DON was produced in carbendazim resistant strains than sensitive ones, but it seemed that carbendazim resistance had no effect on ZEN production in wheat culture.

## 1. Introduction

Members of the *Fusarium graminearum* species complex (FGSC) can infect cereal crops, including wheat (*Triticum aestivum* L.), barley (*Hordeum vulgare* L.), maize (*Zea mays* L.), and rice (*Oryza sativa* L. ) and noncereal crops, including potato (*Solanum tuberosm* L.) and sugar beet (*Beta vulgaris* L.) across the world, causing diseases, including *Fusarium* head blight (FHB) and *Gibberella* ear rot (GER) [1,2]. Besides reducing yield and quality due to the formation of empty grains, the pathogens contaminate infected grains with significant levels of deleterious mycotoxins including trichothecenes and the estrogenic mycotoxin zearalenone (ZEN) [3,4,5]. Trichothecene toxins, such as deoxynivalenol (DON) and nivalenol (NIV), may inhibit eukaryotic protein biosynthesis and have been implicated in a number of human and animal mycotoxicoses [6,7]. Trichothecenes were also proved to be phytotoxic and act as virulence factors on some sensitive plants [8,9]. ZEN is also a major concern, though it has relatively low toxicity and carcinogenicity levels, it can cause hyperestrogenism and reproductive problems in animals [10,11]. Therefore, even low levels of these toxins in raw grain can make them hazardous to human or animal health.

To date, at least 16 phylogenetically distinct species have been identified and described in the FGSC (*F. graminearum* species complex) [12,13,14,15,16]. Most species appear to be restricted to specific geographic regions [17]. In North America and Europe, *F. graminearum* *sensu stricto* (s. str.) is dominant according to the investigation of *Fusarium* spp. composition and population structure [12,13,16]. *F. asiaticum* and *F. graminearum* s. str. were found to be the predominant etiological agents of FHB in Asia, including Japan and Korea, although their distribution varies depending on the sampling sites [18,19,20,21]. In China, the majority of *F. graminearum* s. str. isolates are found in cooler northern regions, and *F. asiaticum* mainly exists in warmer wheat-growing regions where FHB epidemics occur most frequently [22].

FGSC species can produce several mycotoxins and type B trichothecenes are the most common toxic metabolites found in infected cereals [23]. DON is associated with feed refusal, vomiting and suppressed immune functions, and NIV is more toxic to humans and domestic animals than DON [24]. Strains of FGSC usually produce one of the three trichothecene profiles: (i) deoxynivalenol and 3-acetyldeoxynivalenol (3ADON chemotype); (ii) deoxynivalenol and 15-acetyldeoxynivalenol (15ADON chemotype); or (iii) nivalenol, its acetylated derivatives and low levels of DON (NIV chemotype) [25]. These mycotoxin chemotypes showed different geographical distributions and appeared to be associated with toxigenic species. The NIV chemotype has been reported in several countries in Africa, Asia, Europe, and America [16,26,27,28,29], whereas the DON chemotype is more common all over the world.

Various molecular tools have been developed and used for the identification of pathogen and the analysis of the genetic diversity of the natural population. Some house-keeping genes including beta-tubulin, calmodulin, MAT alleles, translation elongation factor 1α (*EF-1α*), H3 histone and the internal transcribed spacers were applied in the phylogenetic analysis. More and more phylogenetic studies of *Fusarium* revealed that *EF-1α* appears as the most useful genetic marker [30,31]. The majority of *Fusarium* species produce toxic secondary metabolites and the key biosynthetic genes are clustered and conserved. Recently, many key secondary metabolite biosynthetic genes in *TRI* and *FUM* clusters were successfully used for species and chemotype identification and phylogenetic researches [32]. It was reported that individual *TRI* genes of *Fusarium* belonging to different trichothecene chemotypes practically reflected the groupings based on the chemotype [12]. Recently, *TRI5* were proven to contain enough intraspecific divergence and could be developed for phylogenetic analyses in the latest report about *F. equiseti* [33].

In recent years, numerous studies have been conducted to examine species and trichothecene chemotypes diversity among FGSC isolates infecting wheat and barley in the middle and lower reaches of the Yangtze River [22,34,35,36,37]. However, few have investigated the diversity of the FGSC obtained from infected maize and rice in this region. In fact, it has been reported that FGSC can also cause significant losses on them in other regions. Since the first report of this fungus on rice [38], it has been known to exist in most of the rice growing regions in the world and there have been several reports of scab on rice in Asian countries, including Korea, Nepal, Japan, and India, in recent years [20,26,39]. The disease usually does not cause heavy damage, but can be severe under conditions favorable to disease development [20]. The fungus can also cause stalk and ear rots of maize with contamination of mycotoxins [40]. Demonstrating the distribution of FGSC and their trichothecene chemotypes in cereal crops will help understanding the relevance between disease and mycotoxin contamination, and thus is critical for developing effective management strategies to control the disease and mycotoxins contamination.

The economic and social impact of plant diseases caused by FGSC highlights the necessity of an effective control strategy. Various strategies have been developed to control FHB or GER and to reduce mycotoxin contamination of crops, with chemical control having an important role in an integrated FHB control program [41]. Carbendazim and other benzimidazole fungicides, which act by inhibiting mitosis, have been shown to be effectively against a variety of plant pathogenic fungi, including most of the ascomycetes and some deuteromycetes, and have been used to control plant dieases over the past three decades [42]. Members of FGSC may become resistant to this fungicide, then, besides the reduced effectiveness, it is very important to know the impact of fungicide resistance on mycotoxin production. There were several previous studies which reported the potential risk of increased contamination of agricultural products due to the appearance of mycotoxigenic isolates resistant to site-specific fungicides.

In this context, a large number of *Fusarium* strains were isolated from wheat, rice and maize in Jiangsu and Anhui provinces, located in lower reaches of Yangtze River, and the objective of this study was to: (i) determine the species identity and trichothecene chemotypes of FGSC isolates; (ii) analyze genetic structure of FHB pathogens from different hosts; (iii) assess the reaction of *Fusarium* isolates to carbendazim; and (iv) measure DON and ZEN production.

## 2. Results and Discussion

### 2.1. Species Composition

**Figure 1:**
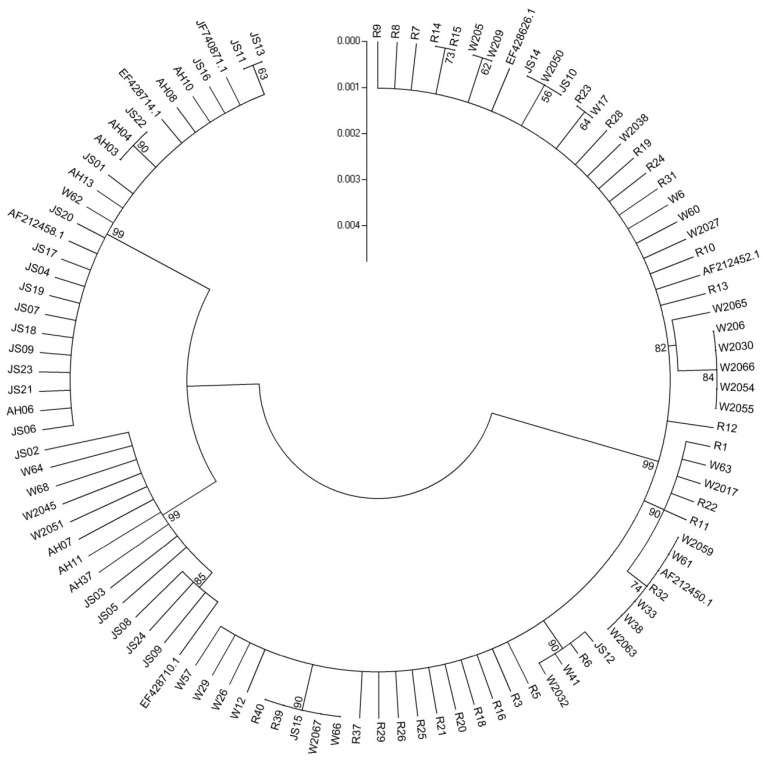
Consensus phylogenetic tree for the isolates in this study, created on the basis of the translation elongation factor 1α (*EF-1α*) sequences. The dendrogram was constructed by the neighbor-joining approach and tested by bootstrapping (10,000 replicates) with a cut-off value of 50%. Three strains of *F. graminearum* (NRRL 28063, 34587 and 52929) and four strains of *F. asiaticum* (NRRL 6101, 26156, 31734, and 34578) were used as references.

**Figure 2:**
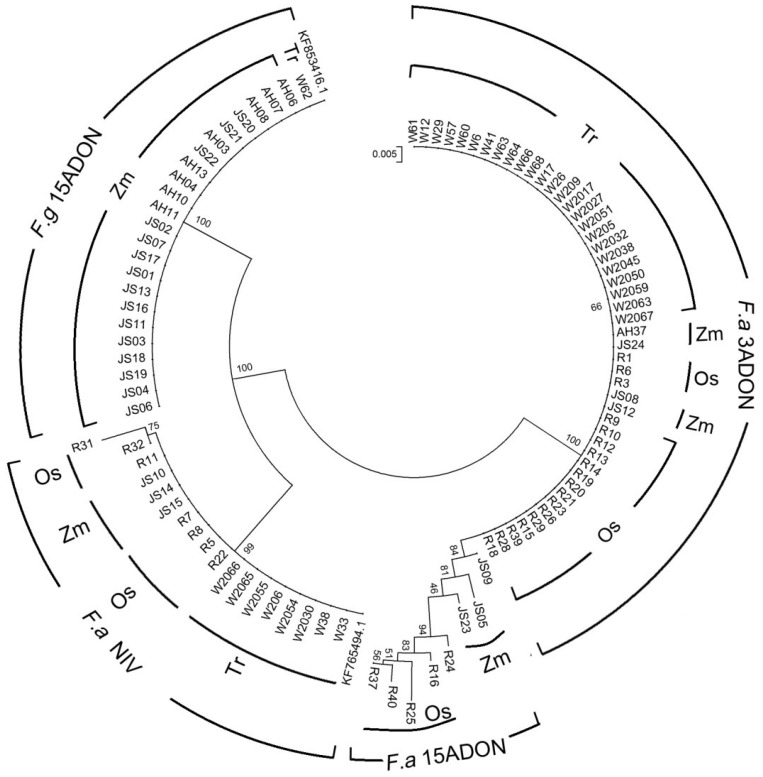
Consensus phylogenetic tree for the isolates in this study, created on the basis of the trichodiene synthase (*TRI5*) sequences. The dendrogram was constructed by the neighbor-joining approach and tested by bootstrapping (10,000 replicates) with a cut-off value of 50%. The *F. graminearum* s. str. 52L (KF853416.1) strain and *F. asiaticum* SCK04 (KF76594.1) strain were used as the reference. Abbreviations used for the chemotype, species and host species: 3ADON:3-acetyldeoxynivalenol; 15ADON: 15-acetyldeoxynivalenol; NIV: nivalenol; F.a: *Fusarium asiaticum*; F.g: *Fusarium graminearum*; Os: *Oryza sativa*; Zm: *Zea mays*; Ta: *Triticum aestivum*.

A total of 97 isolates from 24 sampling sites from two provinces were morphologically identified as members of FGSC according to Leslie and Summerell [40]. These included 33 isolates from maize, 30 isolates from rice and 34 isolates from wheat. PCR analysis with the Fg16F/R primers revealed that each isolate carried a 410-bp or 497-bp DNA fragment. Among the 97 isolates assayed, 73 isolates (75.26%) were identified as *F. asiaticum*, and the remaining 24 isolates (24.74%) belonged to *F. graminearum* s. str. (Table 1, Figure 1 and Figure 2). The vast majority, among the isolates collected from maize, were identified as *F. graminearum* s. str. (69.7%) (Table 1, Figure 1 and Figure 2). In contrast, the composition of FGSC on wheat and rice was different than that was observed on maize. The proportion of *F. asiaticum* isolated from rice and wheat reached 100% and 94.3%, respectively (Table 1; Figure 1 and Figure 2).

**Table 1:** Geographical origin, numbers, species, and trichothecene chemotypes of *Fusarium* populations from rice, maize, and wheat.

Host	County	Numbers	*F. asiaticum*			*F. graminearum*
			3ADON	15ADON	NIV	15ADON
Rice	Rudong	6	4		2	
	Jiangdu	5	3	1	1	
	Dongtai	5	2	2	1	
	Xinghua	5	4		1	
	Baoying	4	2		2	
	Sheyang	5	3	2		
	Subtotal	30	18	5	7	
Maize	Qidong	4	1	1		2
	Huoqiu	3		1	1	1
	Dafeng	4	1		2	1
	Suixi	3		1		2
	Lingbi	4	2			2
	Siyang	5				5
	Sheyang	3				3
	Xinyi	3				3
	Peixian	4				4
	Subtotal	33	4	4	3	23
Wheat	Huoqiu	4	2		1	1
	Rudong	5	3		2	
	Lixin	4	2		2	
	Huaiyuan	4	3		1	
	Xinghua	4	4			
	Sixian	5	4		1	
	Shuyang	4	3		1	
	Tongshan	4	4			
	Subtotal	34	25		8	1

### 2.2. Trichothecene Chemotypes Determination

Trichothecene types were determined using the single and multiplex PCR, and consistent results were obtained for all the isolates. *Fusarium* isolates from three hosts varied in trichothecene chemotypes. The DON chemotype was predominant among isolates from maize (90.9%), while the 3ADON and 15ADON chemotypes reached 12.12% and 78.78%, respectively (Table 1; Figure 2). Based on *TRI5* gene sequence, we also got the same conclusion about the trichothecene genotypes (Figure 2). FGSC species on wheat in this region were predominantly 3ADON producers (73.53%) (Table 1; Figure 2). In rice population, all three trichothecene types were identified, with 18 (60%) being of the 3ADON type, 5 (16.67%) and 7 (23.33%) being of the 15ADON and NIV type, respectively (Table 1; Figure 2).

### 2.3. Phylogenetic Analysis

An internal fragment in *EF-1α* gene of about 620 bp in 97 isolates was amplified and sequenced. *EF-1α* gene sequences of several *F. graminearum* s. str. and *F. asiaticum* strains from GenBank database were downloaded and applied in the phylogenetic study as references. The phylogenetic dendrogram obtained from the partial *EF-1α* gene sequence is shown in Figure 1. All the *F. graminearum* s. str. isolates, including 23 from maize and one on wheat, fell firmly into one cluster. The remaining *F. asiaticum* strains were divided into two clusters. One cluster contained 12 isolates plus one reference NRRL34578 and this cluster were closer to the *F. graminearum* s. str. cluster. The rest of *F. asiaticum* strains and three reference strains were classified into the other cluster. Based on *EF-1α* sequencing, we got the same conclusion with Fg16F/R composition.

The expected 540 bp fragment of *TRI5* gene of 97 isolates were amplified and sequenced. *TRI5* gene sequences of several *F. graminearum* s. str. and *F. asiaticum* strains from GenBank database were downloaded and applied in the phylogenetic study as reference. The phylogenetic dendrogram obtained from the partial *TRI5* gene sequence is shown in Figure 2. All the isolates were grouped into three clusters. The four populations identified by *TRI5* sequence analysis showed significant correlation with both species, chemotype and host of plants. All *F. graminearum* s. str. isolates with 15ADON chemotype were assigned to a cluster and we called it Fg15ADON. Fg15ADON was mostly composed of those isolates on maize. Most *F. asiaticum* strains (64.4%) with 3ADON type were distributed to the cluster named Fa3ADON and these strains collected primarily from rice and wheat. Fa15ADON consist of 15ADON producer of eight *F. asiaticum* strains, including three on maize and five on rice. Fa15ADON and Fa3ADON were classified to one cluster. We named the last cluster FaNIV as those *F. asiaticum* strains produced NIV and they were isolated mainly from wheat rice. FaNIV was closer to Fg15ADON.

### 2.4. Sensitivity of the Fusarium Isolates to Carbendazim

PDA plates containing 1.4 μg/mL carbendazim were used to test all isolates for their sensitivity to this fungicide. 14 isolates were determined to be naturally resistant to carbendazim, including nine isolates from wheat and five isolates from rice. All 33 isolates collected on maize were sensitive to carbendazim.

**Figure 3:**
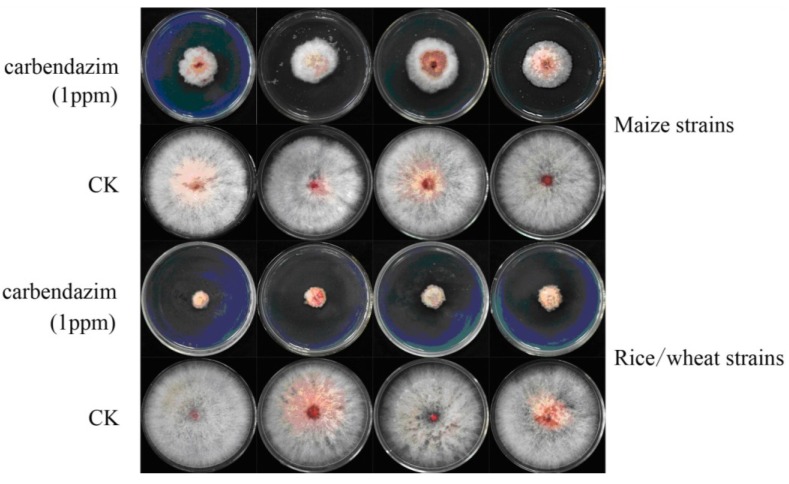
Effects of carbendazim on mycelial linear growth of the isolates from maize, wheat, and rice. All strains were grown for three days on PDA media amended with 0 (CK) and 1 μg/mL carbendazim.

The carbendazim EC_50_ values of sensitive isolates from wheat ranged from 0.47 to 0.77 μg/mL, and the average (± SE) EC_50_ value was 0.6 ± 0.06 μg/mL. The EC_50_ values of the 25 sensitive isolates from rice were 0.58 to 0.79 μg/mL, with a mean of 0.66 μg/mL. Those isolates collected from maize were less sensitive to carbendazim, the inhibition ratio was about 55% by 1 μg/mL carbendazim addition (Figure 3) and EC_50_ values averaged 1.16 ± 0.2 μg/mL. The EC_50_ values of individual isolate to carbendazim were listed in Table S1.

### 2.5. Chemical Analysis of DON and ZEN

**Figure 4:**
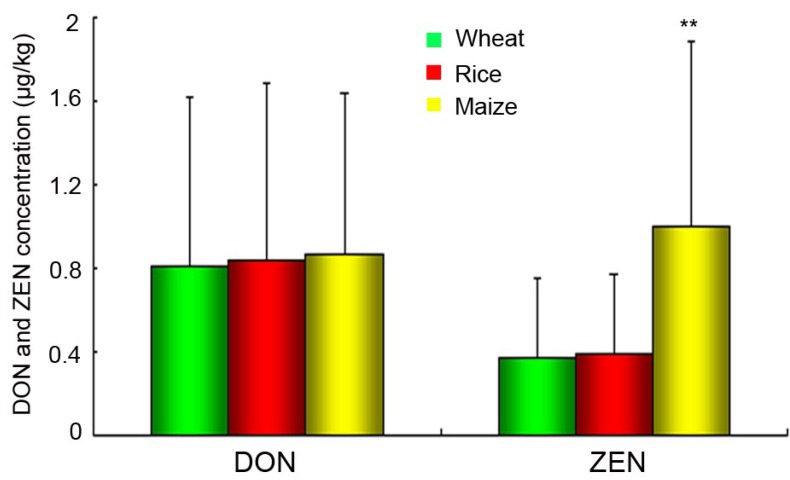
DON and ZEN production of the isolates in this study from different hosts. Means with different letters are significantly different (** *p* < 0.05). Error bars represent standard deviations.

The results of DON and ZEN production of all isolates were acquired. Analysis of variance results showed that the isolates did not differ significantly (*p* < 0.05) in production of DON. The isolates from maize produced significantly (*p* < 0.05) higher amounts of ZEN compared with those on wheat and rice. The average value of ZEN production of individual isolate from wheat, rice, and maize was 0.37, 0.39 and 0.99 μg/kg, respectively (Figure 4).

DON and ZEN production of *Fusarium* isolates were also analyzed based on their sensitivity to carbendazim. More DON (*p* < 0.05) were produced by resistant strains (1.69 ± 0.79 μg/kg) than sensitive strains (0.69 ± 0.71 μg/kg). However, there was no significant difference (*p* < 0.05) in the production of ZEN by resistant (0. 54 ± 0.38 μg/kg) and sensitive strains (0.6 ± 0.71 μg/kg). DON and Zen production of individual isolate to carbendazim were listed in Table S1.

### 2.6. Discussion

Our results indicated FGSC from rice, wheat and maize were genetically different and identified as *F. graminearum* s. str. and *F. asiaticum.* Because many previous reports indicate these two species are predominant in southern China, it is fast and simple to use Fg16F/R for population analysis in our region. In the previous text, we have discussed this matter [43]. Here, all of the isolates were identified by the Fg16F/R primers and analyzed of *EF-1α* and *TRI5* sequence. The *EF-1α* gene has been applied successfully in a variety of researches of *Fusarium* species molecular taxonomy. Recently, an increasing number of studies about secondary metabolite biosynthetic genes as useful methods in the researches of phylogenetic relationships among *Fusarium* species were reported [32]. The corresponding results were obtained and those isolates were accurately divided into *F. graminearum* s. str. and *F. asiaticum.* However, no more clear clades of individual isolate in relation to different host plant was observed as the limited intraspecies divergence of *EF-1α* sequence. Based on these findings, it is likely that *TRI5* can be exploited in phylogenetic researches. The latest instance of advantages of *TIR5*-based phylogeny in *F. equiseti* isolates from Italy and Poland was presented by Stępień *et al.* [32].

When *EF-1α* and *TRI5* phylogenetic tree were compared, three distinct groups of *F. asiaticum* isolates from the *EF-1α* tree seem to be more dispersed when the *TRI5* sequence is analyzed. The comparison of the partial *TRI5* sequences of all isolates studied originating from different hosts allowed to differentiate the chemotype-related groups more clearly than in the case where the *EF-1α* sequences were used. All *F. asiaticum* isolates could be grouped in three genetically distinct clusters, NIV, 3ADON, and 15ADON, respectively. All *F. graminearum* s. str. isolates were classed to 15ADON population. 478 *F. asiaticum* isolates from China and Japan were clustered into 3 populations by Karugia *et al.* [44], with variable number of tandem repeats (VNTR) markers, and each population showed significant correlation with chemotype. Zhang *et al.* [37] divided *F. asiaticum* isolates into two populations with NIV and 3ADON chemotype with the same method. The current study showed similar results. On the other hand, genes from the fumonisins biosynthetic gene cluster were already explored as good targets for phylogenetic studies and useful in revealing the intraspecific polymorphism, specifically correlated with the host plant [32]. As the host has an important influence on species composition, no obvious groups were examined; however, most isolates were dispersed across all groupings with correlation to the host origin and those isolates from the same host are distributed more closely. A lager amount of isolates or new target genes could be applied to study the effect of host on genetic variability. Studies of *F. graminearum* populations have been conducted in different geographic regions; for instance, China [32], Japan [44], the United States [45,46], Germany [47] and Argentina [48]. Populations of *F. graminearum* have high levels of genotypic diversity, which means that recombination exists regularly in *F. graminearum* populations. Great efforts were made on populations from wheat and barley, and the information on *F. graminearum* populations from rice and maize is rare. Furthermore, most studies focus on FGSC members from a single host and there is little research describing variation in original host.

*F. asiaticum* was in the majority of *F. graminearum* clade species on wheat and rice, while *F. graminearum* s. str. predominated in *F. graminearum* clade species on maize. These results were different from that observed in other cereals in China. Zhang *et al.*, [35] and Shen *et al.* [49] suggested that most *Fusarium* isolates collected from scabby wheat were *F. asiaticum.* Recently, a total of 620 isolates from diseased maize ears in 19 provinces throughout China were analyzed and Ndoye *et al.* [50] found that 359 isolates were *F. asiaticum.* The lineage composition of FGSC populations appears to be host and location dependent. In Korea, 75% of the FGSC members from maize belongs to lineage 7 (*F. graminearum* s. str.) [21], more than 80% isolates from rice belongs to lineage 6 (*F. asiaticum*) [20]. In southern America, the occurrence of *F. asiaticum* neatly overlaps with rice-growing areas in Louisiana [29]. They supposed that *F. asiaticum* has a host preference and specificity to rice, and perithecium production typically favors rice straws under warmer conditions. Zhang *et al.* [37] collected the average data of rice, wheat, and maize, and found a strong association between the occurrence of *F. asiaticum* and the predominant crops. *F. asiaticum* has not been detected in northern China where rice is not rotated with wheat in the same field. Therefore, *F. asiaticum* may be more fit only in a rice agro-ecosystem.

The distribution of *Fusarium* species also may be correlated with annual temperature. The relative proportion of *F. graminearum* s. str. and *F. asiaticum* in the population could depend on temperature rather than host preference. Qu *et al.* [22] reported that the vast majority of *F. asiaticum* isolates in China were collected from warmer regions, where the annual average temperature is 15 °C or higher. In Japan [19] and Korea [20], *F. asiaticum* dominated in warmer regions and *F. graminearum* s. str. dominates in cooler regions. However, some reports indicated that temperature may not be the only factor affecting the distribution of *Fusarium* species. Zhang *et al.* [37] reported that the amount of *F. graminearum* s. str. isolates in the southwest region was significantly larger than that in Yangtze River region; nevertheless, the average temperature of southwest region was even a little higher. It is difficult to draw a clear conclusion as there are some other possible explanations including relative humidity, cropping practices, *etc.*

This study developed an overall profile of FGSC from different hosts in the middle and downstream regions of the Yangtze River. All three types of trichothecenes were found but the chemotype composition of *F. graminearum* s. str. and *F. asiaticum* was significantly different. All of the *F. graminearum* s. str. isolates were 15ADON producers, while *F. asiaticum* isolates contained 3ADON and NIV chemotypes. The chemotype composition appeared to be phylogenetic species dependent [35,49]. Furthermore, the distribution of three trichothecenes differed among hosts. In China, *F. asiaticum* strains with NIV chemotypes from maize account for 97% of the total strains, while the same species from wheat mainly produced 3ADON. *F. graminearum* s. str. from both maize and wheat only produce 15ADON [35,50]. In Korea, the majority of *Fusarium* isolates from rice belong to NIV genotype [20]. For Korean isolates collected from maize, all *F. graminearum* s. str. isolates had DON genotype, whereas most *F. asiaticum* isolates had NIV genotype [21]. In contrast, there were no apparent chemotype differences between maize, barley and wheat in some surveys, suggesting that chemotype differences within *F. graminearum* are not particularly critical in host associations. In France, the 15ADON chemotype was predominant among isolates collected from barley, wheat and maize and accounted for 255 of the 294 isolates and only one isolate with 3ADON chemotype was detected [51]. This view cohered with the results observed in South Africa. Boutigny *et al.* [52] reported that 15ADON producers were superior to others. Variations in chemotype composition among FGSC members could be attributed to variation in hosts, geographic area, years, or agricultural practices.

Most of the strains in this study from maize and wheat produce DON, while DON and NIV chemotypes are detected with almost similar proportion in the strains from rice. These results are consistent with the hypothesis that the strains pathogenic to maize and wheat usually are DON producers [21]. In wheat and maize, trichothecene biosynthesis alters strain aggressiveness, with DON-producing strains perceived as more virulent than NIV-producing strains [53]. Trichothecene production is not considered important in aggressiveness towards rice. Maybe the selection for DON producers has occurred within the *F. graminearum* s. str. populations on maize and wheat.

One of the important aims of this study was to explore the influence of original host on the population differentiation and we found a minimal variation in the population structure of *F. graminearum* populations between the three hosts. Population analysis showed strong evidence for high genetic diversity and gene flow and low genetic differentiation among populations from wheat, maize and rice, indicating relatively high genetic exchange among them to maintain a single large population. Previous report found similar results for *F. graminearum* populations from barley, wheat, potato and sugar beet [54]. *F. graminearum* populations overwinter on rice and produce perithecium which is the primary source of infection. In the rotational cropping systems, *F. graminearum* propagules from previous crop residues within surrounding fields may play an important role in genetic exchange between different host populations.

An initial objective of this text was to study the variety of carbendazim sensitivity of *F. graminearum* isolates from different hosts, as there was less report about the effect of host of origin on fungicide efficiency. Only 13 isolates were resistant to carbendazim and all belonged to *F. asiaticum.* These results were in agreement with the previous study in this region [43], which showed that the percentage of carbendazim resistance isolates was higher in *F. asiaticum.* Wang *et al.* [55] documented that colonies of *F. asiaticum* developed normally at a carbendazim concentration of 5.0 μg/mL, indicating that *F. asiaticum* was highly tolerant to carbendazim. In this text, all *F. graminearum* s. str. isolates were sensitive to this fungicide and it seemed that this species was less likely to become resistant to carbendazim. Maize population was less sensitive to carbendazim with higher EC_50_ values, but no resistant isolate was detected in this population. Fungicide was rarely used in maize fields and it can be inferred that increasing use of carbendazim aggrandized the selection pressure and accelerate the occurrence of resistance. As there was diversity in carbendazim resistance of *F. asiaticum* isolates from varied hosts, it can be speculated that fungicide sensitivity was influenced by species and infected host.

More and more researchers paid attention to the use of fungicides and possible effects on mycotoxigenic fungi and mycotoxin production [56]. Researches with laboratory and field isolates have shown that the risk for resistance to fungicides exists in mycotoxin-producing fungi, and there is strong evidence that the inappropriate use of fungicides in the management of crop diseases may reduce sensitivity of certain mycotoxin-producing fungi, and increase toxic contamination of agricultural products. Previous work by D’Mello *et al.* [57] reported that production of the trichothecene mycotoxin 3ADON by *F. culmorum* was affected by the sterol biosynthesis inhibiting fungicide difenoconazole resistance. A higher mycotoxin production (T-2 toxin, 4,15-diacetoxyscirpenol, and neosolaniol) has also been found in a carbendazim resistant strain of *F. sporotrichioides* [58]. Zhang *et al.* [59] found that mutants at codon 167 involved in carbendazim resistance produced significantly more trichothecenes than their wild-type progenitor did. More recently, laboratory mutant strains of *F. verticillioides* and *F. gramineraum* showed that DMI-resistance induced increased persistence of fumonisins and trichothecenes, respectively [60,61]. Similarly, Becher *et al.* [62] found that tebuconazole resistant isolates of *F. graminearum* produced significantly higher levels of NIV compared to the parental sensitive isolate. In addition to the trichothecene NIV, the polyketide mycotoxin ZEN was analyzed and found to exhibit a high variability between different spikelets that were infected by the same fungal strain. Though it is impossible to render statistical evaluation of differences among strains and treatments, it seems that the average ZEN quantity of tebuconazole resistant isolates was higher.

Mycotoxins production of *F. graminearum* field strains varied widely and the effect of carbendazim resistance on mycotoxins production was apparent only when large numbers of resistant and sensitive strains were analyzed. In the present study, a significant larger amount of the DON was found in carbendazim-resistant strains compared to sensitive isolates, while carbendazim resistance did not play an important role in ZEN production. Any attempt to explain the correlation between carbendazim resistance and the increase of DON production in *F. graminearum* would be speculative. Increased efflux is a common mechanism responsible for both resistance and enhanced mycotoxin secretion, but there is no satisfactory explanation for different phenotypes and specificity of resistance at the same time in the case of *F. graminearum*. Zhang *et al.* [59] hypothesized that carbendazim resistance resulted in increased trichothecene production by *F. graminearum* strains through increasing *Tri5* gene expression. There are different synthesis mechanisms of DON and ZEN and it would be helpful to research the effect of mutations at codons involved in carbendazim resistance on some genes relevant to ZEN production. It seems that the relation between *F. graminearum* resistance and increased mycotoxin production remains to be uncovered.

## 3. Experimental Section

### 3.1. Fungal Isolates

**Figure 5:**
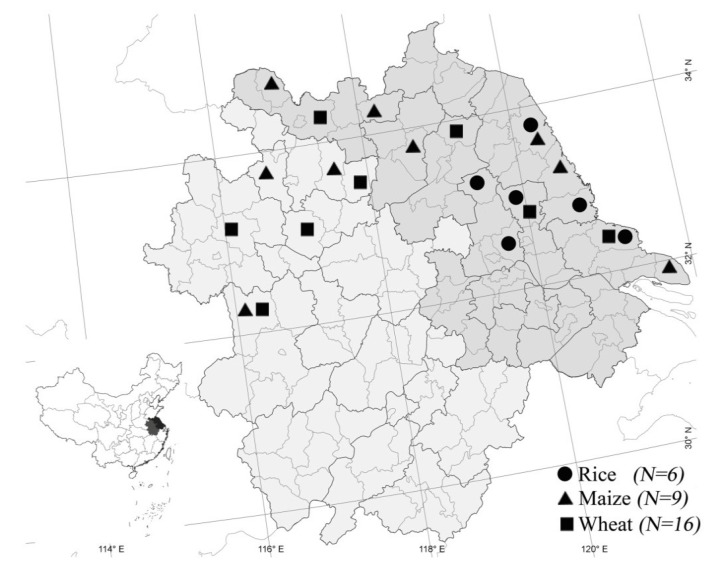
Localization of field samples used in this study to collect FGSC isolates. *N*: number of wheat, barley or maize samples.

Diseased wheat spikes, maize ears and rice seeds were collected near harvest time in 2013 from approximately 24 counties in Anhui and Jiangsu provinces (about 242,600 square kilometers), China (Figure 5). The size of each sampling site was provided in Table 1. All samples were soaked in 5% sodium hypochlorite for 10 min, rinsed in sterile water for 10 min, placed on a sterile filter, transferred on the plate of potato dextrose agar medium (PDA) and then incubated at 25 °C for 4–7 days. Then conidia produced by mung bean broth (MBB) were spread on PDA and a single conidium was isolated. Isolates were maintained on PDA and kept at 4 °C for short-term storage and at −80 °C for long-term storage.

### 3.2. Identification of F. graminearum and Trichothecene Genotype

Each single spore strain grew on PDA plates for 3 days and genomic DNA was extracted from harvested aerial mycelia with a cetyltrimethylammonium bromide (CTAB) method [40].

DNA from all isolates were amplified by PCR with the Fg16F/R primers which produce polymorphic products (400–500 bp) of the *F. graminearum* species complex. The 410-bp DNA fragment was amplified from the isolates belonging to SCAR 1, while the 497-bp fragment was generated from the isolates of SCAR 5 [22,63].

Single and multiplex PCR assays were performed for detection of trichothecene genotypes. Chemotypes of the FGSC isolates were determined using the specific primer pair designed by Li *et al.* [64]. Another two primer sets, Tri303F/Tri303R and Tri315F/Tri315R, targeting the Tri3 gene [28], were used to further characterize DON chemotypes of FGSC. Multiplex PCR assays developed by Wang *et al.* [65] were performed with primer pairs based on the sequence of *Tri11* gene, a key enzyme in the pathway leading to T-2, DON, 3ADON, 15ADON, and NIV biosynthesis in *Fusarium* [66]. They would produce a 279 bp fragment for the 15ADON chemotype, a 334 bp fragment for the 3ADON chemotype, and a 497 bp for the NIV chemotype, respectively.

### 3.3. DNA Sequencing

For sequence analysis, PCR amplification of a partial region of *EF-1α* was carried out with primers EF1T and EF2T [31]. Primers TRI5F and TRI5R were used for the amplification of *Tri5* gene fragments [33].

PCR-amplified DNA fragments were excised from the gel and purified using an AxyPrep DNA Gel Extraction Kit (Axygen, Hangzhou, China) according to the manufacturer’s instructions. Purified PCR products were cloned into pMD T-Vector (TaKaRa, Japan) and sequenced by Shanghai Shenggong Biotechnological Ltd.

### 3.4. Sequence Analysis and Phylogeny Reconstruction

The sequences of the PCR products were aligned and edited with Clustal X 1.81 [67]. Phylogenetic relationships were reconstructed with MEGA4 software package [68] using Maximum Parsimony approach. No gap-containing positions were considered in phylogeny analysis. Bootstrap method used the heuristic search with 1000 replicates.

### 3.5. In Vitro Carbendazim Sensitivity

The sensitivity of all strains to carbendazim was measured as described [69]. Sensitive strains could not grow on PDA plates containing 1.4 μg/mL carbendazim, while resistant strains grew normally on this medium.

### 3.6. Production of ZEN and DON

The steriled wheat culture was inoculated with 10^4^ conidia of individual isolate and incubated at 25 °C for 20 days. Then the wheat culture (10 g) was homogenized in 50 mL mixture of acetonitrile and water (90:10, *v*/*v*) using a tissue homogenizer. The homogenate was kept still for 30 min and filtered through Whatman NO.1 filter paper. The residue was re-extracted once with constant stirring for 30 min, each time with 50 mL acetonitrile-water (90:10, *v*/*v*). The combined filtrate was filtered through a 0.22 μm poly syringe filter before analysis by Liquid chromatography-mass spectrometry.

LC-MS was performed using HPLC (Agilent, Santa Clara, CA, USA) with a Eclipse XDB-C18 (Agilent, Santa Clara, CA, USA), 3.5 μm column, 2.1 × 150 mm. Solvent A was 5 mM ammonium acetate in water, and solvent B was methanol. The program for ZEN and DON analysis was: 0–8 min, 15% B; 8–16 min, 95% B; hold 95% B 8min; 16–24 min linear gradient from 95% B to 15% B; hold 15% B for 1 min. Total runtime was 25 min. The flow rate was 0.2 mL/min. the drying gas nitrogen was set at a flow rate of 12 L/min at 300 °C. The mass spectrometer was operated in positive-ion mode. The instrument was tuned with ZEN and DON standard before analysis for optimum performance.

## 4. Conclusions

Members of FGSC could infect several crops and cause severe plant diseases which are big threat to cereal production and safety. This report contributes to an improved understanding of the species composition, chemotypes and genetic diversity within FGSC across Anhui and Jiangsu provinces in the eastern China. Our findings will be useful in monitoring any future changes in the plant pathogen related to mycotoxin production and for exploring appropriate strategies to control *Fusarium*-caused crop diseases in this region.

## Supplementary Files

Supplementary File 1
