# Supplementary material for: Genetic Relationships, Carbendazim Sensitivity and Mycotoxin Production of the Fusarium Graminearum Populations from Maize, Wheat and Rice in Eastern China
Source: Toxins (Basel). 2014 Aug 4;6(8):2291–309. doi: 10.3390/toxins6082291 (PMC4147583; doi:10.3390/toxins6082291)
Supplement: Supplementary File 1 [file toxins-06-02291-s001.pdf]

Supplementary Information

Table S1. DON, ZEN production and EC_{50} values to carbendazim of individual isolate from different hosts.

strain	host	ZEN	DON	EC50	strain	host	ZEN	DON	EC50	strain	host	ZEN	DON	EC50
JS01	maize	1.0454	0.4368	0.8932	W6	wheat	0.6115	2.1253	0.6462	R1	rice	0.3256	0.3509	0.7719
JS02	maize	0.5874	0.6311	1.0768	W12	wheat	0.2164	0.3632	0.6540	R3	rice	0.4526	0.2958	0.5803
JS03	maize	0.1268	0.3092	1.8349	W17	wheat	1.0289	0.5530	0.5374	R5	rice	0.0768	-	0.6170
JS04	maize	0.3533	1.8636	1.0241	W26	wheat	0.3362	2.1357	3.8015	R6	rice	0.7979	0.9718	0.6363
JS05	maize	0.5494	1.0038	1.2159	W29	wheat	0.1239	0.8006	0.6065	R7	rice	0.5269	-	0.6916
JS06	maize	2.4132	1.5421	0.9864	W33	wheat	0.1064	-	0.6246	R8	rice	0.1312	-	0.6018
JS07	maize	1.1192	0.7889	0.9667	W38	wheat	0.1751	-	0.6189	R9	rice	0.8574	2.2613	4.5645
JS08	maize	0.4614	0.4559	1.0928	W41	wheat	0.0443	1.4758	3.9879	R10	rice	0.0605	0.8236	0.5991
JS09	maize	0.4209	0.7344	0.9972	W57	wheat	1.5223	2.1710	6.3231	R11	rice	0.0445	-	0.7402
JS10	maize	0.2735	-	0.9098	W60	wheat	0.0411	0.1435	0.5357	R12	rice	1.4863	0.2640	0.5794
JS11	maize	1.6855	0.6137	1.1603	W61	wheat	0.2330	0.2647	0.5666	R13	rice	0.3492	1.5065	3.6136
JS12	maize	0.1275	2.7283	1.2737	W62	wheat	0.1343	0.3309	0.4724	R14	rice	0.5689	0.1178	0.6766
JS13	maize	0.1621	0.6346	0.8738	W63	wheat	0.0358	0.1223	0.5086	R15	rice	0.3423	0.0745	0.6561
JS14	maize	0.0515	-	0.8043	W64	wheat	0.6413	3.0520	4.6457	R16	rice	0.0935	0.1223	0.7442
JS15	maize	0.0946	-	1.0766	W66	wheat	0.8539	0.8376	0.4967	R18	rice	0.5633	0.9133	0.7218
JS16	maize	0.3735	0.5250	1.2586	W68	wheat	0.9534	0.8583	0.5705	R19	rice	0.0642	0.1903	0.6233
JS17	maize	2.5753	0.5678	0.9329	W205	wheat	0.0735	0.1776	0.5901	R20	rice	0.0422	0.9068	0.6113
JS18	maize	2.2780	0.1430	1.1418	W206	wheat	0.4640	-	4.1132	R21	rice	0.0485	2.9649	0.6415
JS19	maize	2.0139	0.4184	0.9917	W209	wheat	0.0346	0.8712	4.6457	R22	rice	0.0355	-	0.7963
JS20	maize	0.6479	0.6177	0.8557	W2017	wheat	0.5642	0.1611	0.7684	R23	rice	0.4252	0.6761	0.6338
JS21	maize	0.3337	0.1878	0.9927	W2027	wheat	0.1343	0.3537	0.6166	R24	rice	0.0584	0.1435	0.6662
JS22	maize	0.3523	1.1062	0.8957	W2030	wheat	0.0913	-	0.6018	R25	rice	0.0410	0.2647	0.6105
JS23	maize	0.6106	0.3696	1.1713	W2032	wheat	0.0156	1.7477	0.5967	R26	rice	0.8840	0.3309	0.7360
JS24	maize	0.1406	2.8419	0.8949	W2038	wheat	1.1983	0.0374	0.5806	R28	rice	0.2913	1.4032	3.6260
AH03	maize	2.9538	0.3572	0.8774	W2045	wheat	0.7127	0.8039	3.4934	R29	rice	0.8151	1.4745	3.6724
AH04	maize	0.9843	1.0151	0.8792	W2050	wheat	0.0491	0.1085	0.6213	R31	rice	0.0815	-	0.5898
AH06	maize	2.0982	1.7141	0.8968	W2051	wheat	0.0536	0.3572	0.5772	R32	rice	0.4719	-	0.6932

Table S1. *Cont.*

strain	host	ZEN	DON	EC50	strain	host	ZEN	DON	EC50	strain	host	ZEN	DON	EC50
AH07	maize	1.4308	0.1085	0.7594	W2054	wheat	0.5628	-	0.6651	R37	rice	1.1765	0.1085	0.6165
AH08	maize	0.5188	2.6858	1.1015	W2055	wheat	0.0335	-	0.6334	R39	rice	0.0847	0.3123	0.5802
AH10	maize	2.2239	0.3123	1.1568	W2059	wheat	0.2813	0.4180	5.1661	R40	rice	0.5384	2.6858	5.6145
AH11	maize	1.2973	0.1611	0.7816	W2063	wheat	0.5295	0.7791	0.5687					
AH13	maize	2.4256	0.8357	0.9174	W2065	wheat	0.0832	-	0.6291					
AH37	maize	0.1338	0.2019	0.8215	W2066	wheat	0.6225	-	3.3955					
					W2067	wheat	0.1278	0.0582	0.6853					
